# Supplementary material for: Content-rich biological network constructed by mining PubMed abstracts
Source: BMC Bioinformatics. 2004 Oct 8;5:147. doi: 10.1186/1471-2105-5-147 (PMC528731; doi:10.1186/1471-2105-5-147)
Supplement: Additional File 2 — The original results of the above study (non-essential files are deleted to keep the file size under the limit set by BMC bioinformatics). [file 1471-2105-5-147-S2.bz2 › chilibotAdditionalFile2/dip50/438ID9430724E10383/html/CCR4_CCL22.html]

 


 **CCR4** and **CCL22** 
  
Found 85 abstracts in PubMed, retrieved 50.  
 

 What does Google say? 
 PDF only 
| .edu only 

---

**Interactive relationship** (e.g. stimulation, inhibition, etc)

**Neutral relationship**- Multiple determinants are involved in HIV coreceptor use as demonstrated by  **CCR4**   **CCL22**  interaction in peripheral blood mononuclear cells PBMCs.  Ref: 12429730 J Leukoc Biol, 2002
- MDC  [ **CCL22** ] , which binds  **CCR4** .  Ref: 11507220 J Virol, 2001
- Since the ligand of the TARC and MDC  [ **CCL22** ]  is  **CCR4** , which is specifically expressed on Th2 type T cells, the production of these CC chemokines is likely to play important roles in the T cell and B cell interaction.  Ref: 12715916 J Med Dent Sci, 2003
- The helper Th 2 cell attracting chemokines thymus and activation regulated chemokine TARC and macrophage derived chemokine  [ **CCL22** ]  MDC  [ **CCL22** ]  are ligands for the chemokine receptor  **CCR4** .  Ref: 12645858 J Clin Immunol, 2003
- ... interactions of  **CCR4**  with MDC  [ **CCL22** ]   **CCL22**  or TARC CCL17, CCR8 with I 309 CCL1, and CRTH2 with prostaglandin D 2 play a critical role in the allergen induced recruitment of Th2 cells in the target tissues of allergic inflammation.  Ref: 12009564 Mol Immunol, 2002
- Type 2 T cells have been shown to be preferentially recruited by the chemokines eotaxin CCL11, macrophage derived chemokine  [ **CCL22** ]  MDC  [ **CCL22** ] ,  **CCL22** , and thymus activation regulated chemokine CCL17 through their interaction with CCR3 and  **CCR4** , respectively.  Ref: 12023397 J Immunol, 2002
- However, T cells which are CD4 CD152  **CCR4**  migrate selectively in vitro in response to the chemokine  **CCL22**  specific for the receptor  **CCR4**  produced by the malignant B cells and are always seen amongst the malignant cells in bone marrow and lymph nodes from B CLL patients.  Ref: 12688308 Leuk Lymphoma, 2003
- Moreover,  **CCR4**  and CXCR6 were preferentially induced in T cells activated by mDC  [ **CCL22** ]  and pDC, respectively.  Ref: 12645946 Eur J Immunol, 2003
- Surprisingly, only the  **CCL22**  analogue with the biotin in position K66 constituted a suitable staining reagent for  **CCR4**  positive cells.  Ref: 12857385 J Biomol Screen, 2003
- MDC  [ **CCL22** ]  and were closely surrounded by memory type T cells expressing its receptor,  **CCR4** .  Ref: 12474232 J Pathol, 2003
- The dominance of  **CCL22**  over CCL17 extends to other  **CCR4**  mediated phenomena such as receptor desensitization and internalization and correlates with the peculiar kinetics of  **CCR4**  engagement by the two ligands.  Ref: 12193695 J Immunol, 2002
- Macrophage derived chemokine  [ **CCL22** ]  MDC  [ **CCL22** ]   **CCL22**  is a recently described CC chemokine known to chemoattract the Th2 cytokine producing cells that express the receptor  **CCR4** .  Ref: 11352815 Am J Physiol Gastrointest Liver Physiol, 2001
- Thymus and activation regulated chemokine TARC CCL17 and macrophage derived chemokine  [ **CCL22** ]  MDC  [ **CCL22** ]   **CCL22**  are a pair of CC chemokines known to selectively attract T h 2 type memory T cells via  **CCR4** .  Ref: 12096036 Int Immunol, 2002
- In vitro polarized human Th2 cells preferentially express the chemokine receptors CCR3,  **CCR4** , and CCR8 and migrate to their ligands eotaxin, monocyte derived chemokine MDC  [ **CCL22** ] , thymus and activation regulated chemokine TARC, and I 309.  Ref: 11390417 J Clin Invest, 2001
- T cells expressing CLA and  **CCR4**  were also found at high levels in cutaneous T cell lymphoma lesions along with abundant expression of the two  **CCR4**  ligands TARC CCL17 and MDC  [ **CCL22** ]   **CCL22** .  Ref: 12485447 J Invest Dermatol, 2002
- Recently, macrophage derived chemokine  [ **CCL22** ]  MDC  [ **CCL22** ]   **CCL22** , a CC chemokine, was identified as a selective chemoattractant for CC chemokine receptor 4  [ **CCR4** ]   **CCR4**  expressing cells, in addition to thymus and activation regulated chemokine TARC.  Ref: 11876749 Clin Exp Immunol, 2002
- CC chemokine receptor 4  [ **CCR4** ]   **CCR4**  has been identified as a specific receptor for both thymus and activation regulated chemokine TARC and macrophage derived chemokine  [ **CCL22** ]  MDC  [ **CCL22** ] , and is preferentially expressed on Th2 cells.  Ref: 12757454 Allergy, 2003
- CXCR3 and  **CCR4**  were expressed by dermal CD3 lymphocytes, and their chemokine ligands, IP 10, MIG, TARC, and MDC  [ **CCL22** ] , were up regulated in psoriatic lesions.  Ref: 11310827 Lab Invest, 2001
- Treg cells specifically express the chemokine receptors  **CCR4**  and CCR8 and represent a major subset of circulating CD4 T cells responding to the chemokines macrophage derived chemokine  [ **CCL22** ]  MDC  [ **CCL22** ]   **CCL22** , thymus and activation regulated chemokine TARC CCL17, I 309 CCL1, and to the virokine vMIP I ligands of  **CCR4**  and CCR8.  Ref: 11560999 J Exp Med, 2001
- Three chemokines in particular, CXCL12 SDF 1, CCL17 TARC, and  **CCL22**  MDC  [ **CCL22** ] , recently have been shown to be strong and rapid activators of platelet aggregation and adhesion after their binding to platelet CXCR4 or  **CCR4** , when acting in combination with low levels of primary agonists.  Ref: 12851650 Microcirculation, 2003
- Interestingly, neutralization of macrophage derived chemokine  [ **CCL22** ]  MDC  [ **CCL22** ]  with Ab caused a significant reduction of  **CCR4**  positive T cells within the pancreatic infiltrates and inhibited the development of insulitis and diabetes.  Ref: 12464673 J Clin Invest, 2002
- Fractalkine, SDF 1alpha, RANTES and MDC  [ **CCL22** ]  inhibited the I Ba in CX3CR1, CXCR4, CCR5 and  **CCR4**  expressing G1A1 cells, respectively.  Ref: 11880151 J Neuroimmunol, 2002
- Furthermore, enhanced recruitment of  **CCR4**  bearing cells in NOD mice resulting from transgenic expression of MDC  [ **CCL22** ]  resulted in acceleration of clinical disease.  Ref: 12464673 J Clin Invest, 2002

**Non-interactive relationship** (e.g. studied together, co-existance, homology, etc.)

- In view of the potential importance of  **CCR4**  in skin homing of T cells, we investigated the expression pattern of  **CCR4**  and its ligands TARC CCL17 and MDC  [ **CCL22** ]   **CCL22**  in the peripheral blood and skin of patients with cutaneous T cell lymphoma, a putative malignancy of the skin homing T cells.  Ref: 12485447 J Invest Dermatol, 2002
- Th2 lymphocytes, which are critical for allergy, employ the CC chemokine receptors  **CCR4**  and CCR8 with the ligands thymus and activation regulated chemokine TARC, macrophage derived chemokine  [ **CCL22** ]  MDC  [ **CCL22** ]  and I 309, respectively.  Ref: 11340325 Int Arch Allergy Immunol, 2001
- CONCLUSIONS Our data suggest that Th2 cells, which express  **CCR4**  and its ligands TARC and MDC  [ **CCL22** ] , contribute to the pathogenesis of EP in the lung.  Ref: 12757454 Allergy, 2003
- Macrophage derived chemokine  [ **CCL22** ]  MDC  [ **CCL22** ]   **CCL22**  and  **CCR4**  are involved in the formation of T lymphocyte dendritic cell clusters in human inflamed skin and secondary lymphoid tissue.  Ref: 11290544 Am J Pathol, 2001
- Macrophage derived chemokine  [ **CCL22** ]  MDC  [ **CCL22** ]   **CCL22**  and its receptor  **CCR4**  have been implicated in chronic inflammatory processes and in the homing of monocytes, Th2 cells and regulatory T cell subsets.  Ref: 12225884 J Neuroimmunol, 2002
- Here we show the involvement of the macrophage derived chemokine  [ **CCL22** ]  MDC  [ **CCL22** ]   **CCL22**  and its specific receptor CC chemokine receptor 4  [ **CCR4** ]   **CCR4**  in the formation of T cell DC clusters.  Ref: 11290544 Am J Pathol, 2001
- In this study, we also found that MDC  [ **CCL22** ]  activity on Mo Mphi in this GN was at least partly dependent on a new variant of  **CCR4** .  Ref: 12651599 Am J Pathol, 2003
- The coreceptor activity was sensitive to  **CCR4**  specific antibodies and to the  **CCR4**  specific chemokine ligand macrophage derived chemokine  [ **CCL22** ]  MDC  [ **CCL22** ]  chemokine ligand 22  [ **CCL22** ]   **CCL22** .  Ref: 12429730 J Leukoc Biol, 2002
- BACKGROUND Macrophage derived chemokine  [ **CCL22** ]  MDC  [ **CCL22** ]  is a Th2 type chemokine and its receptor CC chemokine receptor 4  [ **CCR4** ]   **CCR4**  is preferentially expressed on Th2 cells.  Ref: 12670721 J Dermatol Sci, 2003
- Type 1 chemokines CXC chemokine ligand CXCL 9 and CXCL10 monokine induced by IFN gamma and IFN gamma inducible protein of 10 kDa CXCR3 ligands, and type 2 chemokines CC chemokine ligand CCL 11 eotaxin CCR3 ligand, CCL17 thymus and activation regulated chemokine  **CCR4**  ligand and  **CCL22**  monocyte derived chemokine  **CCR4**  ligand were readily detectable in the plasma of most participants.  Ref: 12407016 Int Immunol, 2002
- Thymus and activation regulated chemokine and macrophage derived chemokine  [ **CCL22** ] , ligands for  **CCR4** , were measured by ELISA in JRA synovial fluid, JRA plasma, adult rheumatoid arthritis synovial fluid, and normal plasma.  Ref: 11359851 J Immunol, 2001
- Cells transfected with gpCCR4 migrated toward the human and murine orthologues of the  **CCR4**  ligands, macrophage derived chemokine  [ **CCL22** ]  and thymus and activation regulated chemokine.  Ref: 11724798 J Biol Chem, 2002
- Our data suggest that the MDC  [ **CCL22** ]   **CCR4**  system plays an important role in the formation of T cell DC clusters both in inflamed skin and LNs.  Ref: 11290544 Am J Pathol, 2001
- These facts suggest possible involvement of two  **CCR4**  specific ligandsi.e., thymus and activation regulated chemokine TARC and macrophage derived chemokine  [ **CCL22** ]  MDC  [ **CCL22** ] , in the pathogenesis of bronchial asthma via recruitment of TH2 cells to inflammatory sites.  Ref: 11929424 Allergy, 2002
- The aims of this study were to examine the expression of three recently described potent T lymphocyte chemoattractants, PARC pulmonary and activation regulated chemokine, macrophage derived chemokine  [ **CCL22** ]  MDC  [ **CCL22** ] , and I 309, the MDC  [ **CCL22** ]  receptor  **CCR4** , and T lymphocyte activation markers, CD25, CD26, CD62L, CD71, and CD30, and to correlate them with the counts of CD3 T lymphocytes in the conjunctiva of patients with VKC.  Ref: 12234902 Br J Ophthalmol, 2002
- Supernatants from malignant cells cultured in the presence of sCD40L promote the migration of activated T cells that express  **CCR4** , the common specific receptor for MDC  [ **CCL22** ]  and TARC.  Ref: 11468146 Blood, 2001
- Immunohistochemically, MDC  [ **CCL22** ]  cells and  **CCR4**  cells were mainly located within the T cell DC clusters both in the dermis of inflamed skin and the T cell area of LNs.  Ref: 11290544 Am J Pathol, 2001
- We analyzed the functional role of macrophage derived chemokine  [ **CCL22** ]  MDC  [ **CCL22** ] , a potent mononuclear cell chemoattractant, during the progression of anti glomerular basement membrane GBM antibody Ab GN, a model of crescentic GN in the WKY rat, and whether the effects of MDC  [ **CCL22** ]  were dependent on its receptor  **CCR4** .  Ref: 12651599 Am J Pathol, 2003
- which express high levels of  **CCR4**  with  **CCL22**  caused down modulation of endogenous  **CCR4**  but had no significant effect on HIV 1 entry, suggesting that  **CCR4**  may not be used as an entry coreceptor.  Ref: 12429730 J Leukoc Biol, 2002
- Among  **CCR4**  ligands, skin vessels endothelial cells present C C chemokine ligand CCL 17 but not  **CCL22**  consistent with CCL17 involvement in T lymphocyte arrest on endothelial cells.  Ref: 12193695 J Immunol, 2002
- In this study, we investigate the effects of VIP PACAP on two chemokines, i.e. CXCL10 IP 10 acting on CXCR3 expressed on activated Th1 cells, and  **CCL22**  MDC  [ **CCL22** ]  acting on  **CCR4**  and 8 expressed on activated Th2 cells.  Ref: 12446011 J Neuroimmunol, 2002
- By contrast, expression of  **CCR4**  and responsiveness to  **CCL22**  is rapidly induced at the early stages of both Th1 Th2 cell development.  Ref: 11981813 Eur J Immunol, 2002
- This was associated with the expression of their agonistic receptor CXCR3, while mRNA for TARC and MDC  [ **CCL22** ] , and their receptor  **CCR4**  were not detected.  Ref: 11912011 J Dermatol Sci, 2002
- METHOD Conjunctival biopsy specimens from 11 patients with active VKC, and eight control subjects were studied by immunohistochemical techniques using a panel of monoclonal and polyclonal antibodies directed against PARC, MDC  [ **CCL22** ] , I 309,  **CCR4** , CD25, CD26, CD62L, CD71, and CD30.  Ref: 12234902 Br J Ophthalmol, 2002
- Supernatants from the basal, but not apical, side of polarized epithelial cells induced a MDC  [ **CCL22** ]   **CCL22**  dependent chemotaxis of  **CCR4**  positive T cells.  Ref: 11352815 Am J Physiol Gastrointest Liver Physiol, 2001
- There was a significant correlation between the number of  **CCR4**  CD4 T cells and the levels of TARC, MDC  [ **CCL22** ] , and IL 5 in BALF of patients with EP.  Ref: 12757454 Allergy, 2003
- These supernatants induced the migration of activated CD4, CD40L T cells expressing the  **CCL22**  receptor,  **CCR4** .  Ref: 11981828 Eur J Immunol, 2002
- Expression of the  **CCR4**  specific ligands MDC  [ **CCL22** ]  and TARC is strongly upregulated on airway epithelial cells upon allergen challenge, suggesting an involvement of this receptor ligand axis in the regulation of lymphocyte recruitment into the asthmatic bronchi.  Ref: 11390417 J Clin Invest, 2001
- Importantly, mature dendritic cells preferentially attract Treg cells among circulating CD4 T cells, by secretion of  **CCR4**  ligands CCL17 and  **CCL22** .  Ref: 11560999 J Exp Med, 2001
- Here, we demonstrate that MDC  [ **CCL22** ]  and  **CCR4**  mRNAs are expressed in the central nervous system CNS of mice developing relapsing remitting and chronic relapsing forms of experimental autoimmune encephalomyelitis EAE.  Ref: 12225884 J Neuroimmunol, 2002
- Furthermore, we detected strong signals for  **CCR4** , TARC, and MDC  [ **CCL22** ]  in ATL skin lesions by RT PCR.  Ref: 11861261 Blood, 2002
- Reverse transcriptase polymerase chain reaction analysis revealed high levels of mRNA expression for MDC  [ **CCL22** ]  and  **CCR4**  in inflamed skin and neck lymph nodes LNs, but not in normal skin.  Ref: 11290544 Am J Pathol, 2001
- In contrast to naive T cells, primed T cell arrest was largely inhibited by pertussis toxin, neutralization of the CC chemokine, macrophage derived chemokine  [ **CCL22** ]   **CCL22** , or by desensitization of the  **CCL22**  receptor,  **CCR4** .  Ref: 11673480 J Immunol, 2001
- These data support the role of  **CCR4** , of its ligands MDC  [ **CCL22** ]  and TARC, and of CCR8 in the pathogenesis of allergen induced late asthmatic responses and suggest that these molecules could be considered as targets for therapeutic intervention.  Ref: 11390417 J Clin Invest, 2001
- Accumulation of  **CCR4**  expressing CD4 T cells and high concentration of its ligands TARC and MDC  [ **CCL22** ]  in bronchoalveolar lavage fluid of patients with eosinophilic pneumonia.  Ref: 12757454 Allergy, 2003
- and 4 an efficient migration of fresh ATL cells to the  **CCR4**  ligands, TARC CCL17 and MDC  [ **CCL22** ]   **CCL22** , in chemotaxis assays.  Ref: 11861261 Blood, 2002
- At 4 and 8 weeks after reconstitution of Rag 2 mice the mRNA levels of IP 10, MCP 1, MDC  [ **CCL22** ] , MIG, TARC, RANTES,  **CCR4**  and CCR5 were significantly increased prior to the appearance of macroscopic lesions.  Ref: 11465103 Eur J Immunol, 2001
